# Compensatory saccade in the vestibular impaired monkey

**DOI:** 10.3389/fneur.2023.1198274

**Published:** 2023-09-14

**Authors:** Yoshiko Kojima, Leo Ling, James O. Phillips

**Affiliations:** ^1^Department of Otolaryngology-HNS, University of Washington, Seattle, WA, United States; ^2^National Primate Research Center, Virginia Merrill Bloedel Hearing Research Center, University of Washington, Seattle, WA, United States

**Keywords:** vestibular, monkey, compensatory saccade, covert saccade, overt saccade, animal model

## Abstract

**Introduction:**

Loss of the vestibulo-ocular reflex (VOR) affects visual acuity during head movements. Patients with unilateral and bilateral vestibular deficits often use saccadic eye movements to compensate for an inadequate VOR. Two types of compensatory saccades have been distinguished, covert saccades and overt saccades. Covert saccades occur during head rotation, whereas overt saccades occur after the head has stopped moving. The generation of covert saccades is part of a central vestibular compensation process that improves visual acuity and suppresses oscillopsia. Understanding the covert saccade mechanism may facilitate vestibular rehabilitation strategies that can improve the patient’s quality of life. To understand the brain mechanisms underlying covert saccades at the neural level, studies in an animal model are necessary. In this study, we employed non-human primates whose vestibular end organs are injured.

**Methods:**

We examined eye movement during the head-impulse test, which is a clinical test to evaluate the vestibulo-ocular reflex. During this test, the monkeys are required to fixate on a target and the head is rapidly and unexpectedly rotated to stimulate the horizontal semi-circular canals.

**Results:**

Similar to human subjects, monkeys made compensatory saccades. We compared these saccades with catch-up saccades following a moving target that simulates the visual conditions during the head impulse test. The shortest latency of the catch-up saccades was 250 ms, which indicates that it requires at least 250 ms to induce saccades by a visual signal. The latency of some compensatory saccades is shorter than 250 ms during the head impulse test, suggesting that such short latency compensatory saccades were not induced visually. The peak velocity of the short latency saccades was significantly lower than that of longer latency saccades. The peak velocity of these longer latency saccades was closer to that of visually guided saccades induced by a stepping target.

**Conclusion:**

These results are consistent with studies in human patients. Thus, this study demonstrates, for the first time, compensatory covert saccades in vestibular impaired monkeys.

## Introduction

The vestibulo-ocular reflex (VOR) plays a critical role in gaze stabilization. Patients with vestibular deficit cannot maintain their gaze on the target during head movement because the gaze moves with the head movement away from the target. This unstable gaze causes oscillopsia, an illusion of an unstable visual world (1–4). The head-impulse test reveals this unstable gaze (3). During this test, the subject is asked to fixate on a target and the head is rapidly and unexpectedly rotated to stimulate the semi-circular canals in the plane of the rotation. Optimally, the peak velocity of head movement should be >120°/s and the amplitude should be 10–20° (5, 6).

In normal subjects, for example, when the head turns to the right, the VOR rotates the eyes to the left, so the gaze is stabilized on a stationary target. In patients with a vestibular disorder, head rotations toward the side of a vestibular lesion do not produce fully compensatory eye movements so the gaze moves with the head. The target image slips on the retina during head movement. This retinal slip causes low visual acuity and oscillopsia. After the head movement, the patient makes a saccade, called an “overt saccade,” to acquire the target again.

To minimize the retinal slip, patients learn to make a new type of saccade, called a “covert saccade.” Covert saccades start during the head movement so the duration of retinal slip is shortened and the displacement of the visual image is reduced. Moreover, because vision is suppressed during a saccade (7), there is little retinal slip during the covert saccade. A patient who has acquired covert saccades shows an improvement in the Dizziness Handicap Inventory score, higher visual acuity, and less oscillopsia (6). Thus, learning to generate covert saccades is important for improving the patient’s quality of life. Despite this, little is known about their neural basis.

Several studies have examined the characteristics of the covert saccade in human subjects. (1) Most patients in the early stages of vestibular disorders make only overt saccades. After vestibular rehabilitation, the majority of patients acquire covert saccades. As patients acquire the covert saccades, their vestibular symptoms decrease (2, 3, 6, 8). These results indicate that the covert saccade is “learned” in patients with vestibular disorders. (2) The latencies of covert saccades are less than ~150 ms, and can be as low as ~70 ms (9–11), which is much less than volitional saccades to new visual targets [~200 ms; (12)]. This suggests that covert saccades can be triggered by non-visual cues such as neck proprioceptive signals or residual labyrinthine input etc. (3) Covert saccade latency is shorter when the patient’s head turns are active rather than passive (9, 13, 14). Also, a predictable passive head movement triggers covert saccades earlier than unpredictable head movements (9, 15). These results suggest that covert saccade latencies are influenced by internal signals such as efference copies of head movement and higher-order prediction signals. (4) The amplitude of covert saccades is more compensatory when the residual VOR gain is higher, indicating that better labyrinthine information gives a better estimate of head movement (9). (5) The plot of the saccade peak velocity versus amplitude, so-called “main sequence,” indicated that the velocity of covert saccades is lower than the velocity of visually-guided saccades of the same size (14), implying that the neural mechanisms that generate covert saccades are different from those that generate visually-guided saccades (16).

To study the neural mechanisms for covert saccades, an animal model is required. To the best of our knowledge, no such model exists. Fortunately, there is an animal model of vestibular dysfunction which has been used in our laboratory in the development of a vestibular prosthesis in non-human primates (17–19). In this study, we examined in non-human primates with vestibular dysfunction whether they make covert saccades like human patients. We also examined their main sequence to evaluate whether it exhibits similar characteristics with that of human saccades.

## Materials and methods

All experiments were performed in accordance with the Guide for the Care and Use of Laboratory Animals and exceeded the minimal requirements recommended by the Institute of Laboratory Animal Resources and the Association for Assessment and Accreditation of Laboratory Animal Care International. All the procedures were evaluated and approved by the local Animal Care and Use Committee of the University.

### Surgery and training

Two male *Macaca mulatta* monkeys (E and D) participated in this study. We implanted each monkey with fixtures to affix their head to the primate chair and a scleral search coil (20) to measure eye position in space. After the monkeys had recovered from the surgery, we trained them to track a small visual target in a dimly lit, sound-attenuating booth. The target was a 0.3° laser spot projected onto a cylindrical projection screen via two computer-controlled orthogonal mirror galvanometers. The primate chair was embedded in a servo-controlled multiaxis rotator (Actek, Seattle, WA). A custom program running in Spike2 (Cambridge Electronic Design, Cambridge, United Kingdom) controlled target movement, chair rotation, and the monkey’s reward via a CED Power1401 computer interface.

The monkey sat in a primate chair with its head restrained. We measured eye position with the electromagnetic search coil method (21). We rewarded the monkeys with applesauce for keeping their gaze within ±2° windows around the horizontal and vertical positions of the target spot for at least 0.5 s. Once they had learned to fixate the target spot, we trained them to make visually-guided saccades to a stepping spot that moved to random locations on the tangent screen within a ± 18° radius off straight-ahead. We delivered an applesauce reward (~0.16 ml per dollop, ~200 ml/h) by a pump (Masterflex tubing pump, Cole-Parmer, Vernon Hills, United States) every 2 s regardless of the amplitude, direction, or timing of the saccade, as long as it landed within the ±2° window surrounding the target. The targeting saccade was required to occur within 0.6 s of the target step and the subsequent fixation had to be maintained for at least 0.3 s for a reward to be given.

Under anesthesia, we implanted a vestibular prosthesis in the right ear of a rhesus monkey. This may have reduced vestibular function in the implanted ear, although this was not apparent in subsequent testing (17). Briefly, responses to the en-bloc velocity step testing, sinusoidal rotation testing (across a range of 0.05–2.0 Hz, ±10°), and constant peak velocity (across a range of 0.01–1.0 Hz, 80°/s) were normal. After full recovery from the implantation surgery, a series of transtympanic gentamicin injections were performed in monkey E, (18). Briefly, two holes were made in each tympanic membrane, and gentamicin (40 mg/ml, 0.2 ml/injection) was injected through one hole until the solution flowed from the other hole. This procedure was repeated 4 times (18). We suspect monkey D had an innate loss of the left horizontal semicircular canal because we could not find the left semicircular canal during the prosthesis surgery. Since the left horizontal semicircular canal had been dysfunctional enough by this loss and/or surgical damage, we did not inject gentamicin. Table 1 provides dates of these procedures and experiments.

**Table 1:** Dates of the procedures and experiments.

Monkey E	
Jun 22, 2011	Prosthesis implant surgery, right ear
March 27, 2013	Gentamicin injection, left ear
April 3, 2013	Gentamicin injection, right ear
April 19, 2013	Gentamicin injection, left ear
April 25, 2013	Gentamicin injection, right ear
July 31, 2019	VOR gain measurement in this study
July 31, 2019	First experiment in this study
April 25, 2022	Last experiment in this study
Monkey D	
September 8, 2021	Prosthesis implant surgery, right ear
November 17, 2021	Prosthesis implant surgery, left ear (no implant)
February 22, 2022	Prosthesis explant surgery, right ear
July 5, 2022	VOR gain measurement in this study
July 5, 2022	First experiment in this study
February 2, 2023	Last experiment in this study

**Figure 1:**
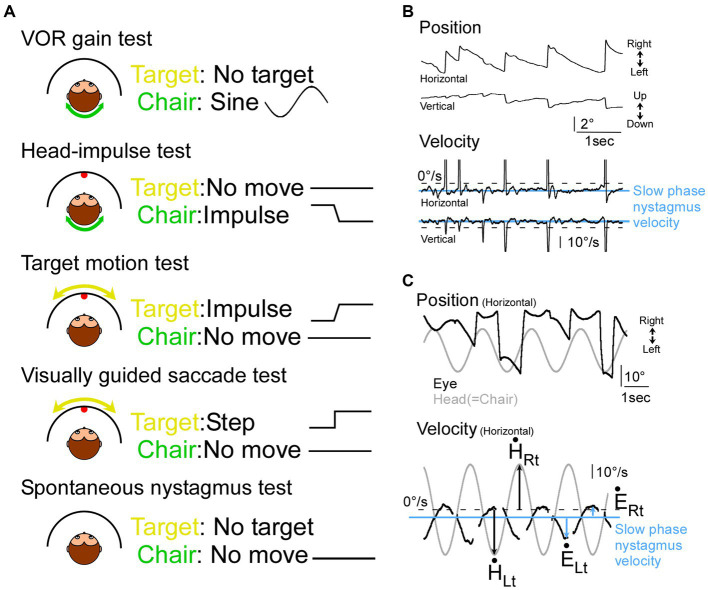
Illustration of tests and representative eye movement traces in monkeys with vestibular impairment. **(A)** Illustration of 5 tests in this study. **(B)** Nystagmus in monkey D during the spontaneous nystagmus test. **(C)** Records of head (grey) and eye movement (black) during sinusoidal VOR test in monkey E during the VOR test. Top panel, eye and target position. Bottom panel, eye and target velocity. Blue horizontal lines: slow phase nystagmus velocity, black arrows: peak head velocity, blue arrows: peak eye velocity from the slow phase nystagmus velocity.

We measured the VOR gain in complete darkness. The chair was moved sinusoidally at 0.5 Hz frequency and ± 10° amplitude. Once we confirmed the VOR gain had been reduced (see Result, Figure 1), we started the head-impulse test training (3, 22). The animals were required to fixate on a stationary target during chair rotation. We started with a slow horizontal rotation of the chair (25°/s). To make sure the animal stayed comfortable and safe, we did not exceed 150°/s head velocity and 2000°/s/s head acceleration and deceleration during the chair motion.

### Experimental procedures

We conducted 43 experiments in 2 monkeys, 18 from monkey E and 25 from monkey D. We performed 5 tests, the VOR gain test, the head-impulse test, the target motion test, the visually guided saccade test, and spontaneous nystagmus test (Figure 1A).

#### VOR gain test

We measured the VOR gain independently by rotating the animal sinusoidally in complete darkness. The chair was moved at a frequency of 0.5 Hz and with an amplitude of ±10° (one session for each monkey).

#### Head-impulse test

In the head-impulse tests (336 ± 140 impulses/session, 43/43 sessions), the animal fixated a stationary target while the chair was rotated at 100°/s peak velocity (900°/s/s peak acceleration) and ± 15° amplitude in the horizontal plane (rightward and leftward). The inter trial interval was randomized between 4, 5, or 6 s. In 10 of 43 experiments (monkey E), we also rotated the chair at 150°/s (2000°/s/s) and ± 15° amplitude in the horizontal plane.

#### Target motion test

In this test (74 trials, 1 session, monkey E), we used the same command signal that was used to move the chair in the HIT. But in this test, we inverted that signal and used it to drive the motion of the target spot. In this way, we reproduced the relative motion of a stationary target during the HIT. The purpose of this test is to evaluate the contribution of pursuit to the HIT response.

#### Visually guided saccade test

In this test (210 ± 115 trials/session, 27/43 sessions), the animal followed a target stepped 3 to 12° every 1°. The chair remained stationary during this test.

#### Spontaneous nystagmus test

In this test (18 ± 26 saccades/session, 30/43 sessions), the animal was placed in the dark while the chair remained stationary. Without a target to fixate the animals were exhibiting a spontaneous nystagmus.

### Data analysis

We digitized eye, target, and chair position signals at 1 kHz using the Power1401 data acquisition/controller hardware (Cambridge Electronic Design, Cambridge, United Kingdom). Data were saved to a hard drive for later analysis.

A custom program running in Spike2 analyzed the saved data. It detected the occurrence of a saccade when eye velocity exceeded 150°/s within 50–800 ms after a program issued a target jump or initiation of chair movement and marked saccade onset and end when vector eye velocity exceeded or fell below 100°/s, respectively. The program measured saccade amplitude, peak velocity and duration, and the target distance before each saccade. The saccade attributes, target, and chair positions were exported to MATLAB (MathWorks, Natick, United States). Saccades whose initial eye positions differed from initial target positions by >5° were not analyzed.

To calculate the VOR gain, we first measured the slow phase velocity in the spontaneous nystagmus test session, estimating the background drift in the dark (monkey E: 4.6°/s leftward, 3.9°/s upward. Monkey D: 6.1°/s leftward, 5.4°/s upward; Figures 1B,C “Velocity” panel, blue line). Next, we measured the peak eye velocity during a slow phase in the VOR gain test session (Figure 1C “Velocity” panel, blue arrows). We also measured the corresponding peak head velocity (Figure 1C “Velocity” panel, black arrows). Finally, we calculated a gain value for leftward head rotation (=rightward eye movement) by dividing the peak rightward eye velocity (Figure 1C “Velocity” panel, blue arrow at “Ė_Rt_”) by the peak head velocity to the left (Figure 1C “Velocity” panel, black arrow at “_Lt_”). Similarly, a gain value for rightward head rotation (=leftward eye movement) was calculated by dividing the peak leftward eye velocity (Figure 1C “Velocity” panel, blue arrow at “Ė_Lt_”) by the peak head velocity to the right (Figure 1C “Velocity” panel, black arrow at “_Rt_”; (17)). The VOR gain was established by averaging 14 such measurements.

**Figure 2:**
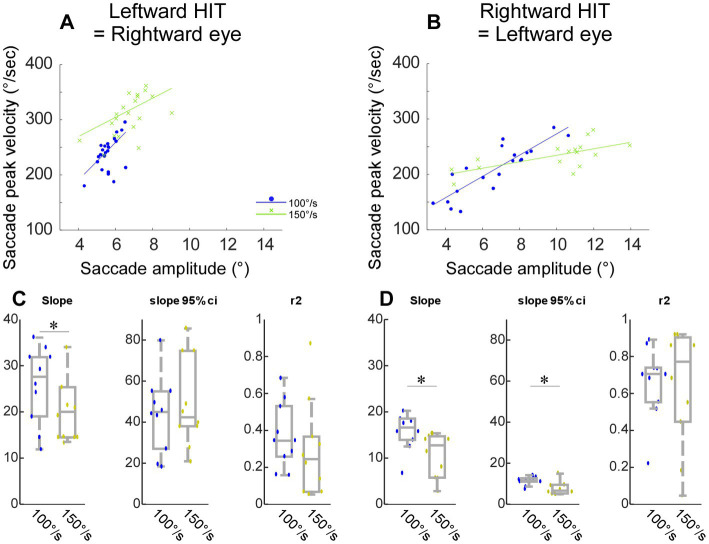
Representative saccades during the head-impulse test **(A–C,G–I)** and target motion test **(D–F,J–L)** from monkey E. **(A–F)** Rightward eye movements. **(G–L)** Leftward eye movements. Black traces: eye movement. Grey traces: target movement relative to the head (=inverted chair movement in **A**,**B**,**G**, and **H**). X axis: Time 0 is at the computer command to rotate the chair **(A,B,G,H)**, or to move the target **(D,E,J,K)**. Y axis: negative value indicated leftward. **(B,E,H,K)** Velocity traces. **(C,F,I,L)** Saccade latency distribution in one session [*n* = 26 **(C)**, 31 **(F)**, 34 **(I)**, 42 **(L)**].

**Figure 3:**
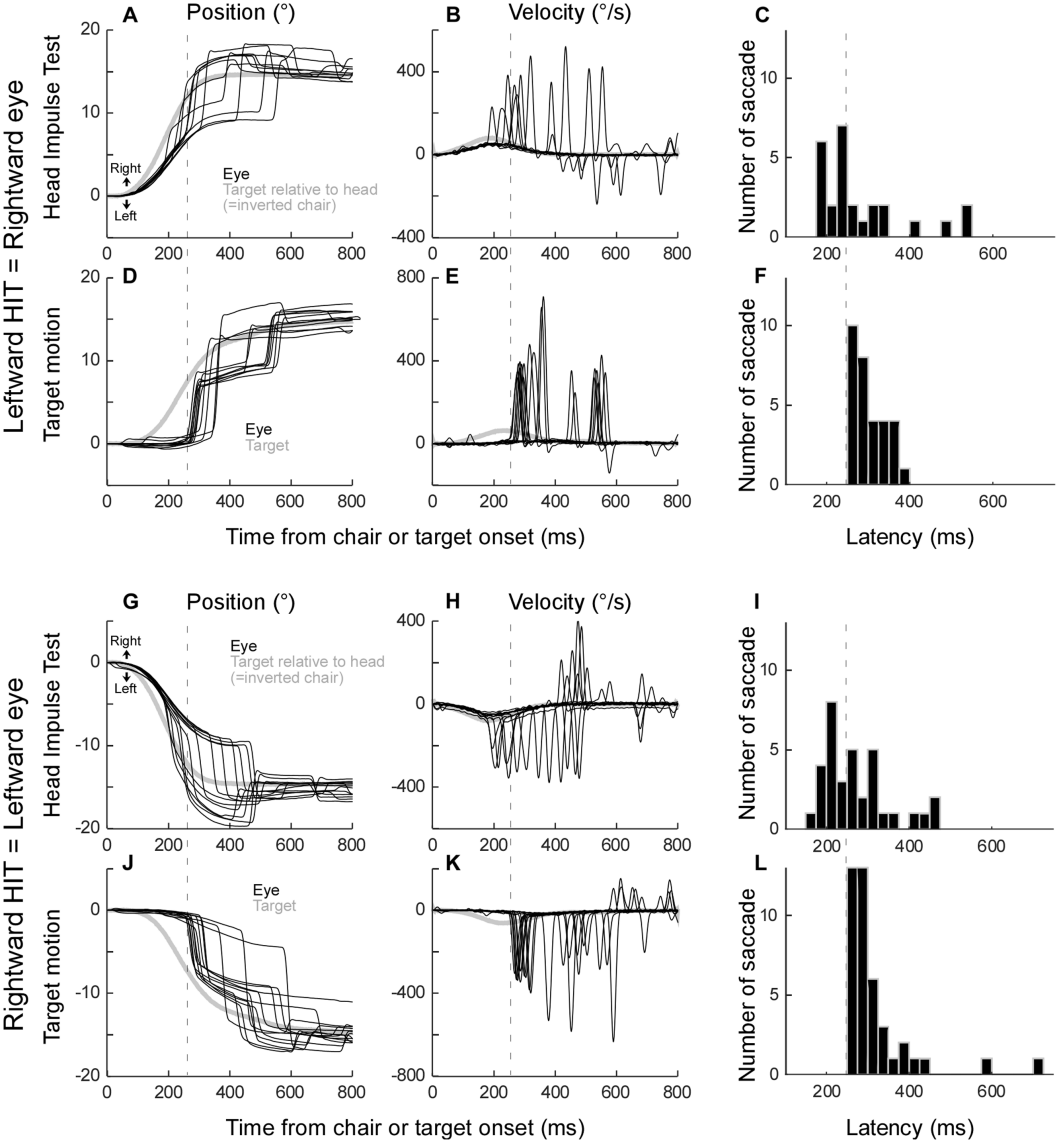
Catch-up saccade latency for each target speed (target motion test).

To demonstrate the eye movements during the head impulse test, we showed eye movement traces and saccade latencies (Figure 2). To evaluate the lack of effect of target movement speed on saccade latency in the data of the target motion test, we used one-way ANOVA (Figure 3).

**Figure 4:**
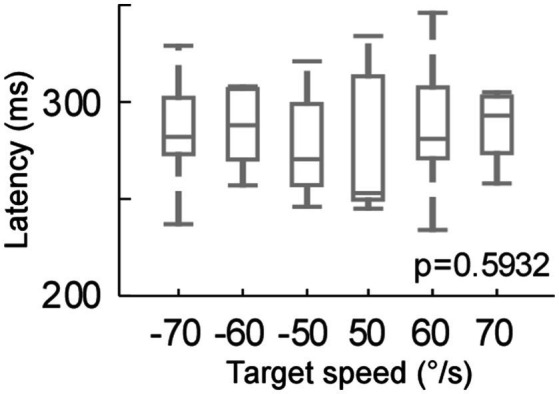
Relationship between saccade peak velocity and saccade amplitude in 4 types of saccade. **(A,E)** A representative experiment of rightward saccades **(A)** and leftward saccades **(E)** from monkey D. Circles, dots, or triangles are single saccades, lines are regression lines. **(B–D,F–H)** Summary of all experiments from monkey E. **(I,J)** Summary of all experiments from monkey D. **(B–D,I)** Rightward saccades. **(F–H,J)** Leftward saccades. Comparison of slope [**(B,F)**, left panels of **(I,J)**], 95% confidential interval of the slope [**(C,G)**, middle panels of **(I,J)**], and *r*-square [**(D,H)**, right panels of **(I,J)**] of the regression line. Box plot (grey) and swarm plot (color dot). *Significant. HIT-NV, putative non-visual saccade during head impulse test (100°/s). HIT-PV, putative visual saccade during head impulse test (100°/s). VGS, visually guided saccade during visually guided saccade test. Nystagmus, quick phase nystagmus during spontaneous nystagmus test.

**Figure 5:**
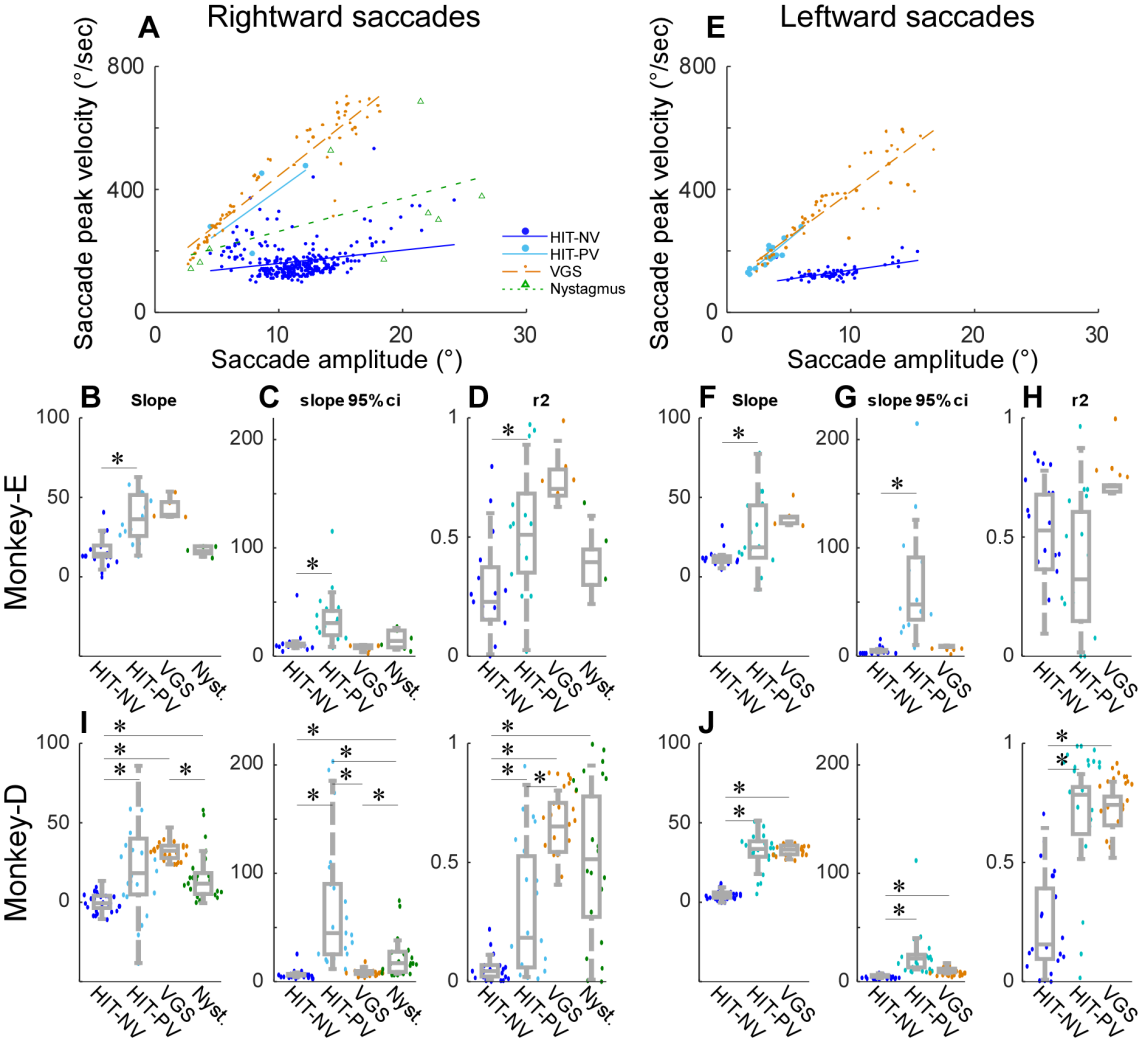
Comparison between HIT-NV recorded during the 100°/s and 150°/s chair rotation in monkey E (head impulse test). **(A-D)** Same organization as in Figures 4A–H.

To compare the characteristics of each saccade type, that is, putative non-visual and visual saccades during head impulse test, visually guided saccade, and quick phase nystagmus (Figure 4) and putative non-visual saccades during 100°/s and 150°/s head impulse tests (Figure 5), we plotted the main sequence, namely saccade peak velocity against saccade amplitude, and fitted them with a linear regression line. We then compared the slope of each saccade type across all 43 experiments (Wilcoxon signed rank tests). We considered a variable to be significant only when the value of p was less than 0.05.

## Results

In this study, we examined whether the non-human primate with vestibular impairment makes covert saccades during the head impulse test (HIT). We will first confirm the impairment of the VOR in the two non-human primates that were examined in this study. Next, we will show eye movements during the HIT (100°/s). To address concerns about saccade latency and the retinal slip (see below for details), we performed two control experiments. Then, to verify that the saccades in this study were similar to the covert saccades in previous studies of human patients, we will compare the main sequence of these saccades with those of different saccade types. Finally, we will compare the main sequence during HIT using either 100°/s or 150°/s to see whether the HIT speed affects the saccades.

### Confirmation of VOR impairment (spontaneous nystagmus and VOR gain tests)

Both monkeys had spontaneous nystagmus. Figure 1B shows a representative eye movement during the nystagmus test session in monkey D. The slow phase nystagmus velocity was 4.6°/s leftward, and 3.9°/s upward in monkey E and 6.1°/s leftward and 5.4°/s upward in monkey D (Figure 1B). Figure 1C shows a representative eye movement during the VOR gain test session in Monkey E. The VOR gain was 0.23 ± 0.039 to leftward head rotation (=rightward eye movement) and 0.45 ± 0.058 to rightward head rotation (=leftward eye movement). The other monkey’s VOR gain was 0.21 ± 0.038 to leftward head rotation and 0.76 ± 0.074 to rightward head rotation. Thus, we confirmed that both monkeys had vestibular impairment.

### Saccade during the head impulse test (head-impulse test-100°/s)

The eye movement response to a leftward head-impulse test (=rightward saccade) is shown in Figure 2A. In this representative example, the chair was rotated 15° to the left, while the target remained stationary, so the monkey needed to make an eye movement to the right in order to keep fixated on the stationary target. Residual VOR moved the eye initially, however, because the VOR gain was low, the distance between the eye and the target increased as the chair movement progressed. To catch the target, saccades were executed with variable latencies. Figure 2B shows the velocity traces of these trials. The latency distribution of all 26 trials in this session are in Figure 2C.

### Control experiment – visually induced saccade (target motion test)

Since the duration of the chair movement was 300 ms in this experiment, saccades with less than 300 ms latency were considered covert saccades by definition (3, 4, 22). However, because 300 ms was longer than the duration of head movements in previous human studies (2, 3, 6, 9, 22, 23), we were concerned that these saccades with less than 300 ms latency might contain not only the so-called covert saccades but also overt saccades. Therefore, we differentiated the saccades in a different way: whether the saccade could be induced visually or not.

To induce saccades visually without vestibular stimulus, we moved the visual target with dynamics similar to those of the chair movement. The chair remained stationary in this condition. Figure 2D shows representative target movement and respective catch-up saccades. The relative target movement was identical to that during the head-impulse test in Figure 2A (grey line). The monkey made a catch-up saccade in order to fixate on the target; however, its latency was longer than those saccades with chair rotation. The latency distribution of all 31 trials in this session is shown in Figure 2F.

The shortest latency of the catch-up saccade in the target movement condition was 250 ms (dashed lines in Figures 2C,F). This suggested that the saccade whose latency was more than 250 ms during head impulse test could be induced visually, while the saccades with less than 250 ms latency during the head impulse test might be induced by a non-visual signal. Thus, we split the saccades during the head impulse test into 2 groups, the saccades with less than 250 ms latency as putative non-visually induced saccades and the saccades with more than 250 ms latency as putative visually induced saccades.

Figures 2G–I show the eye movement response to rightward head-impulse test (=leftward saccade). Similar to the leftward head-impulse test, the representative saccades were executed with variable latencies (Figures 2G,H). The latency distribution of all 34 trials in this session is in Figure 2I. Figures 2J–L show the target motion trials. Again, the latency was longer than those of saccades with chair rotation and the shortest latency of the catch-up saccade in the target motion trials was 250 ms (Figure 2L, *n* = 42).

### Control experiment – target motion speeds (target motion test)

Because the residual VOR moved the eye initially in the head impulse test, the visual input, i.e., retinal slip, was slower in the head impulse test than the target motion condition. Therefore, we compared the effect of retinal slip speed on catch-up saccade latency (Figure 3). We moved the target at 50, 60, and 70°/sec right- and leftwards (positive and negative values, respectively). The target speed did not have a significant effect on the saccade latency (One-way ANOVA, *p* = 0.59). Thus, the significant latency difference during the head impulse test and the target motion condition could not be explained by the difference in retinal slip velocity.

### Comparison with another saccade types (head-impulse test-100°/s, visually guided saccade test, and spontaneous nystagmus test)

To verify that the characteristics of the saccades in monkeys were similar to that of previous human studies, we examined the correlation of saccade peak velocity as a function of saccade amplitude, namely the saccade main sequence. Figure 4A shows a representative experiment (monkey D) to compare the main sequence plots between these putative non-visual (HIT-NV) and putative visual (HIT-PV) rightward saccades (=leftward HIT). The correlation between saccades peak velocity and saccade amplitude for the non-visual saccades (regression line slope = 4.3, slope *p* = 4.03 × 10^−5^, *r*^2^ = 0.05) was significantly weaker (*p* = 0.012) than for visual saccades (regression line slope = 29.0, slope *p* = 0.034, *r*^2^ = 0.43; Figure 4A). Thus, the main sequences of non-visual and visual saccades were different, suggesting that the generation of these 2 types of saccades might be different.

Because the putative visual saccades might be induced visually, we compared its main sequence with that of visually guided saccades (collected during the visually guided saccade test, Figure 1A; VGS, Figure 4A) which are induced by a stepping target. The rightward visually guided saccade exhibited a stronger correlation than the visual saccades (regression line slope = 31.8, slope *p* = 1.00×10^−15^, *r*^2^ = 0.87). We also plotted the main sequence of quick-phase saccades during nystagmus (collected by the spontaneous nystagmus test, Figure 1A). The quick phase saccades exhibited an intermediate slope between the visual and non-visual saccades (regression line slope = 10.8, slope *p* = 0.013, *r*^2^ = 0.29).

Figures 4B–D summarizes the properties of the regression line among the 4 types of rightward saccades, that is, putative non-visual (HIT-NV) and putative visual saccade (HIT-PV) during head impulse test, visually guided saccade (VGS), and quick phase nystagmus, across all experiments from monkey E. We compared the 4 types of saccades to each other, so there were 6 comparisons (Wilcoxon signed rank tests with Bonferroni Correction for 6 times repetition, significant level: *p* < 0.0083). Figure 4B compares the slopes. HIT-NV exhibited a significantly lower slope than HIT-PV (*p* = 0.00064). The remaining 5 comparisons were not significantly different.

Figure 4C compares the 95% confidence interval of the slopes. HIT-NV exhibited a significantly smaller interval than HIT-PV (*p* = 0.0072). Figure 4D compares the r-square of the slopes. HIT-NV exhibited a significantly smaller correlation than HIT-PV (*p* = 0.00044).

Figures 4F–H summarizes 3 types of leftward saccades across all experiments from monkey E (no quick-phase nystagmus saccades to the left). Similar to the rightward saccades, HIT-NV exhibited a significantly lower slope and 95% confidence interval than HIT-PV (slope: *p* = 0.017, Figure 4F confidence interval: *p* = 0.00044, Figure 4G).

Figures 4I,J summarize all experiments from monkey D. This monkey showed significant differences in more comparisons. The consistent result from 2 monkeys and 2 directions were; 1, the slope of HIT-NV was lower than that of HIT-PV. 2, the slope of HIT-PV was not different from that of VGS. 3, the 95% confidence interval of HIT-NV was lower than that of HIT-PV.

Since the deficits of VOR were different between the monkeys and directions, we examined these factors on the slope of the main sequence. The median and IQR of the slope of rightward and leftward HIT-NV were 13.4 ± 7.7 and 11.6 ± 4.0, respectively, in monkey E (Figures 4B,F) (*p* = 0.085, Wilcoxon signed rank tests), and − 1.2 ± 9.1 and 4.4 ± 3.5, respectively, in monkey D (Figures 4I,J; *p* = 4.03 × 10^−5^, Wilcoxon signed rank tests). The *p* values of 2-way ANOVA for the slope of HIT-NV were 1.46×10^−12^ (the factor monkeys) and 0.24 (the factor directions). Thus, there was a significant difference between the monkeys but no significant difference between the directions in HIT-NV saccades.

The median and IQR of the slope of rightward and leftward HIT-PV were 35.5 ± 25.4 and 18.8 ± 33.1, respectively, in monkey E (Figures 4B,F; *p* = 0.16, Wilcoxon signed rank tests), and 14.3 ± 28.4 and 33.1 ± 9.6, respectively, in monkey D (Figures 4I,J; *p* = 0.044, Wilcoxon signed rank tests). The p values of a 2-way ANOVA for the slope of HIT-PV were 0.19 (the factor monkeys) and 0.51 (the factor directions). Thus, in HIT-PV saccades, there were no significant differences between monkeys and directions. Finally, the median and IQR of the slope of rightward and leftward VGS were 39.1 ± 8.8 and 37.4 ± 4.7, respectively, in monkey E (Figures 4B,F; *p* = 0.031, Wilcoxon signed rank tests), and 30.4 ± 7.3 and 32.7 ± 5.5, respectively, in monkey D (Figures 4I,J; *p* = 0.25, Wilcoxon signed rank tests). The p values of a 2-way ANOVA for the slope of VGS were 2.14×10^−7^ (the factor monkeys) and 0.47 (the factor directions). Thus, for VGS, there was a significant difference between the monkeys but no significant difference between the directions.

### Saccades with faster head impulse (head-impulse test-100°/s and 150°/s)

Figures 5A,B show a representative experiment to compare HIT-NV that occurred during the 100°/s and 150°/s chair rotation. For both rightward (Figure 5A) and leftward saccades (Figure 5B), the slope of the regression line fitted to the main sequence for the 150°/s was lower than that in the 100°/s. Across 10 experiments from monkey E, the slope was significantly lower in the 150°/s than 100°/s [*p* = 0.03 for rightward (Figure 5C), 0.02 for leftward (Figure 5D)]. Thus, the saccade HIT-NV that occurred during the 150°/s had more extreme properties as regards to their main sequence relationships.

## Discussion

This study demonstrated that monkeys with vestibular dysfunction made compensatory saccades. Also, we found that the short-latency saccade during the head impulse test exhibited a distinct characteristic compared to the other types of saccade, such as long latency saccade during head impulse, visually guided saccade, and the quick phase of nystagmus.

Traditionally in human studies, saccades during the head impulse test are divided into two types, covert and overt saccades, by a criterion based on head movement duration. Covert saccades occur during head rotation, whereas overt saccades occur after the head has stopped moving (3, 22). Because we had to rotate the primate chair slower than during the head impulse test in the human subjects (see below), we were reluctant to use this traditional criterion separating covert and overt saccade in this study. We, therefore, isolated the short latency saccades during the head impulse test based on the latency of catch-up saccade induced by retinal slip. This criterion allowed us to consider putatively the short latency saccade as a non-visually induced saccade. Note that this short latency saccade cannot be an anticipatory saccade because we randomized the intertrial interval so the monkeys could not anticipate the initiation of the chair rotation. Also note that we are not proposing to dissociate covert and overt saccades by catch-up saccade latency in human subjects as we did in this study. We used this strategy simply as an accommodation for our slow head impulse test in our experiments. We did so to make sure that the animal stayed comfortable and safe during the experiment. This slower rotation should also be beneficial for future neurophysiological experiments which envisage to record the neural activity during the head impulse test.

Are the short-latency saccades in monkeys similar to the covert saccades in human patients? The latency of our short latency saccade was less than 250 ms, whereas those of human covert saccades was less than ~150 ms. However, our chair took 85.3 ms to move the first 1° measured from the computer command. Therefore, the actual latency of our short latency saccade could be shorter, ~165 ms, closer to the human covert saccades of ~150 ms. Furthermore, the main sequence results are consistent with the study of human patients; that is, the peak velocity of short latency saccades was lower than that of visually guided saccades of the same amplitude (9).

Another difference between this study in monkeys and human studies was the subject’s neck movement. In this study, the monkey’s body and head were rotated en-bloc, so the neck movement was minimized. While, in human experiments, the body remained stationary when the head rotated, so the neck moves. Thus, the contribution of the neck proprioceptive signal is smaller, if any, in this monkey study than in the human studies and the contribution of the cervico-ocular reflex is reduced (9). As reported previously, many sensory signals can contribute to inducing covert saccades, such as residual vestibular signal (9), a visual signal (23), proprioceptive signal, anticipatory signal (9, 13–15), and other signals that we do not yet know, suggesting that the brain mechanism of covert saccade could be variable. The short latency saccade in this study could be a simple version of the covert saccade, since the contribution of neck proprioceptive and timing anticipation were limited.

## Conclusion

To the best of our knowledge, this is the first study that has examined the compensatory saccade in monkeys with reduced vestibular function. The short latency saccades in this study showed similar properties to human covert saccades. This simple version of the covert saccade in the monkey could be a useful model to study the basis of the neural mechanism underlying the covert saccade.

## Data availability statement

The raw data supporting the conclusions of this article will be made available by the authors, without undue reservation.

## Ethics statement

The animal studies were approved by University of Washington Animal Care and Use Committee. The studies were conducted in accordance with the local legislation and institutional requirements.

## Author contributions

All authors designed the experiments, interpreted the data, contributed to the article, and approved the submitted version. YK and LL performed the experiments. YK conceived the experiments and analyzed the data, prepared the figures, and wrote the manuscript. LL and JP edited the manuscript.

## Funding

This study was supported by National Institute of Health (NIH) grants EY023277 and EY033760 (YK), R21 DC-018083 (Newlands/JP), R01 DC014002 (Rubinstein), HHS-N-260-2006-00005-C (JP), UW Royalty Research Fund A148416 (YK) and made possible by NIH grants OD010425 (P51 for WaNPRC), and Vision Research Core (P30EY001730 for UW).

## Conflict of interest

The authors declare that the research was conducted in the absence of any commercial or financial relationships that could be construed as a potential conflict of interest.

## Publisher’s note

All claims expressed in this article are solely those of the authors and do not necessarily represent those of their affiliated organizations, or those of the publisher, the editors and the reviewers. Any product that may be evaluated in this article, or claim that may be made by its manufacturer, is not guaranteed or endorsed by the publisher.
